# Ketogenic Diet Combined with Moderate Aerobic Exercise Training Ameliorates White Adipose Tissue Mass, Serum Biomarkers, and Hepatic Lipid Metabolism in High-Fat Diet-Induced Obese Mice

**DOI:** 10.3390/nu15010251

**Published:** 2023-01-03

**Authors:** Jiabao Yan, Cuiru Ren, Yunlong Dong, Jibran A. Wali, Hongjie Song, Ying Zhang, Hengrui Zhang, Guangning Kou, David Raubenheimer, Zhenwei Cui

**Affiliations:** 1 Centre for Sport Nutrition and Health, Centre for Nutritional Ecology, School of Physical Education (main campus), Zhengzhou University, Zhengzhou 450001, China; 2 Charles Perkins Centre, The University of Sydney, Sydney, NSW 2006, Australia; 3 School of Physical Education, Zhengzhou University of Light Industry, Zhengzhou 450002, China

**Keywords:** ketogenic diet, aerobic exercise, lipid metabolism, obesity

## Abstract

Obesity is a serious public health issue worldwide. Growing evidence demonstrates the efficacy of the ketogenic diet (KD) for weight loss, but there may be some adverse side effects such as dyslipidemia and hepatic steatosis. Aerobic exercise is a widely recognized approach for improving these metabolic markers. Here we explored the combined impacts of KD and moderate aerobic exercise for an 8-week intervention on body weight and fat loss, serum biomarkers, and hepatic lipid metabolism in a mouse model of high-fat diet-induced obesity. Both KD and KD combined with exercise significantly reduced body weight and fat mass. No significant adverse effects of KD were observed in serum biomarkers or hepatic lipid storage, except for an increase in circulating triglyceride level. However, aerobic exercise lowered serum triglyceride levels, and further ameliorated serum parameters, and hepatic steatosis in KD-fed mice. Moreover, gene and protein expression analysis indicated that KD combined with exercise was associated with increased expression of lipolysis-related genes and protein levels, and reduced expression of lipogenic genes relative to KD without exercise. Overall, our findings for mice indicate that further work on humans might reveal that KD combined with moderate aerobic exercise could be a promising therapeutic strategy for obesity.

## 1. Introduction

Obesity has become one of the major public health issues worldwide, which affected over 650 million people in 2020 according to the World Health Organization [1]. It is a significant risk factor for many chronic diseases, including type 2 diabetes, insulin resistance, hypertension, hyperlipidemia, cardiovascular diseases, osteoarthritis, and some cancers [2,3], and has substantial economic implications for society [4]. In general, obesity is caused by a long-term excess of energy intake relative to energy expenditure [5]. Multiple factors, including genetic susceptibility, dietary habits, physical activity, and environmental characteristics influence the development of obesity [6,7]. Intervention strategies mainly focus on nutrition, exercise, bariatric surgery, and medication [6], to reduce the level of energy intake, increase energy expenditure or reduce energy absorption. Since all these approaches have limitations, ranging from poor compliance to minimal long-term efficacy to very high costs of treatments, finding effective and safe ways to lose weight and improve obesity outcomes remains a research priority.

In recent years, carbohydrate-restricted diets, in particular, the ketogenic diet (KD), have received increasing attention in academic and public health settings [8,9]. KD is distinguished by its specific ratios of macronutrients, involving a high amount of fat (sometimes as high as 90% of calories), moderate protein content, and very low amounts of carbohydrates (typically < 10% of daily energy) [10,11]. Initially used in the treatment of epilepsy [12], recent evidence shows that the advantages of KD in weight loss for obesity are significant [8,13,14]. The KD mimics a fasting state, triggering physiological changes in substrate utilization from glucose-based energy metabolism to the metabolism of ketone bodies derived from lipid stores [15].

Some of the effects of KD on metabolic health and body fat in humans and rodents remain controversial. For example, previous studies on rodents have shown that weight loss on KD was accompanied by a significant reduction in lean mass [16,17]. However, Roberts et al. showed that 6 weeks of KD feeding reduced body weight in rats without affecting skeletal muscle protein synthesis [18]. In addition, some previous findings on humans have shown that KD intervention lowered body weight as well as fat mass in obese patients [19,20]. Additionally, despite its beneficial effects on weight loss in the short term, long-term exposure to KD may have some potential adverse effects. For example, a considerable number of studies have shown that KD may result in dyslipidemia, hepatic lipid accumulation, hepatic steatosis, fibrosis as well as inflammation [11,16,21]. Other studies have reported that KD can improve hepatic fat accumulation and can even be used as an effective treatment for the reversal of nonalcoholic fatty liver disease (NAFLD) [22,23,24]. Thus, it is still unclear whether KD induces adverse effects on hepatic lipid metabolism when used for weight loss by obese patients.

An important and under-explored question concerns the potential roles for modulators of lipid metabolism during KD diets used in obesity. A well-known modulator of lipid metabolism is aerobic exercise training, which numerous studies have reported can increase fat utilization, reduce hepatic lipid accumulation, and improve hepatic steatosis [25,26]. In this study, we aimed to explore the impact of the combination of KD and exercise for an 8-week intervention on hepatic lipid accumulation as well as markers of overall metabolic health and lipid profile in a mouse model of obesity (high-fat diet-induced obesity). We hypothesized that in sedentary obese mice, KD may induce dyslipidemia and hepatic steatosis but combining KD with exercise would mitigate these effects while still yielding the benefits of KD such as weight loss and reduced adiposity. We compared body mass, energy intake, substrate metabolism, serum parameters (i.e., serum lipids, aspartate aminotransferase, and alanine aminotransferase), white adipose tissue characteristics (i.e., visceral fat mass and epididymal adipocyte cell size), and markers of liver biology (i.e., lipid accumulation, lipogenesis and lipolysis-related gene and protein expression patterns) in obese mice exposed to different dietary and/or exercise interventions.

## 2. Materials and Methods

### 2.1. Animals

Eight-week-old male C57BL/6J mice were obtained from the Beijing HFK Laboratory Animal Company (Beijing, China), and were maintained at the Centre for Sport Nutrition and Health of Zhengzhou University. Mice were housed in ventilated cages under a controlled environment (22 ± 2 °C, 12 h light/12 h dark cycle), and were allowed to acclimatize for a week before formal experimental intervention. Food and water were supplied ad libitum and their intake was measured daily throughout the experiment, and the body weight of mice was recorded once a week. The animal experimental procedures were conducted following the guidelines of the NIH for the Care and Use of Laboratory Animals and were approved by the Life Sciences Institutional Ethics Review Board of Zhengzhou University (Approval number: ZZUIRB 2021-145).

### 2.2. Obese Mouse Model and Dietary Intervention

After one week of acclimatization on a regular chow diet, mice were randomly divided into two groups: a control group and a high-fat group, which were provided a standard chow diet (AIN-93G) and a high-fat diet (60% kcal fat, H10060, Beijing HFK Bioscience Co., Ltd., Beijing, China), respectively [27]. After 12 weeks of the induction of the high-fat diet, mice in the high-fat group that achieved the criteria for obesity (more than 20% of the mean body weight of control group mice) were randomly divided into four groups (n = 8 per group): a high-fat diet sedentary group (HFD), a high-fat diet plus aerobic exercise group (HFD + EX), a ketogenic diet sedentary group (KD), and a ketogenic diet plus aerobic exercise group (KD + EX). Mice in both the HFD and HFD + EX groups were continued on the high-fat diet containing 60% fat energy (H10060), while the KD and KD + EX groups were provided a ketogenic diet containing 90% energy from fat (XTKD01, Xietong Pharma Co., Ltd., Nanjing, China). Both the high-fat diet and ketogenic diet were representative of experimental diets commonly used in rodent studies and were based on the formulas of the original high-fat diet (D12492) and ketogenic diet (D10070801) (Research Diets, New Brunswick, NJ, USA), respectively [28,29]. At the same time, mice in HFD + EX and KD + EX groups were subjected to exercise training, which was performed after acclimatization to HFD or KD intervention for one week. Mice in the control group (CON) were continued to feed the standard chow diet (AIN-93G). The dietary composition of the experimental diets is detailed in Tables S1 and S2.

### 2.3. Exercise Training Protocol

The exercise intervention was performed on a motorized experimental treadmill with 0% gradient (XR-PT-10B, Shanghai, China). Initially, mice were familiarized with the treadmill running for two weeks. Over the first week, mice were acclimated to the treadmill at a speed of 5 m/min, 15–20 min/day, for three days a week. During the following week, the duration and running speed were gradually increased. Mice continued to adapt to running at 6 m/min for 10 min of warm-up, then 20 min of the main exercise at 10 m/min, and 10 min of cool down at 6 m/min. Subsequently, formal exercise was carried out on the treadmill for a total of 8 weeks. This consisted of 5 min of warm-up at 6 m/min, followed by 50 min of the main exercise at 12 m/min, and 5 min of cool-down at 6 m/min performed for 5 consecutive days per week. The exercise intensity was moderate, approximately 58–75% of VO_2max_ [30,31].

### 2.4. Indirect Calorimetry

The assessment of the metabolic status of mice was performed with a Columbus CLAMS system (Columbus, OH, USA). At the beginning of the experiment, mice were acclimated to the system for 24 h. The animals were maintained under the 12 h light/dark cycle with free access to food and water. The rate of O_2_ consumption (VO_2_), rate of CO_2_ production (VCO_2_), respiratory exchange ratio (RER, VCO_2_/VO_2_), and the level of energy expenditure were measured over the next 24 h to monitor the experimental process. The activity level of mice in all groups was evaluated by the automatic recording of revolutions of the running wheel.

### 2.5. Histological Analysis

Liver tissue and epididymal white adipose tissue (eWAT) samples harvested from mice were initially fixed in 4% of paraformaldehyde, then dehydrated in graded ethanol, and embedded in paraffin. The paraffin tissue blocks were cut into 5–8 μm sections, then stained with hematoxylin and eosin (H&E Staining). For Oil-red O staining, liver tissues that had been fixed in 4% of paraformaldehyde were dehydrated in 20% and 30% sucrose solutions and embedded in optimal cutting temperature compound. Thereafter the frozen sections were used for Oil-red O staining. The H&E and Oil-red-O-stained sections were viewed with an optical microscope at an original magnification of 200×. The processes of the histological experiment mentioned above were conducted by Servicebio Laboratory (Wuhan, China).

### 2.6. Serum Biochemical Analysis

Blood samples were collected and serum was isolated by centrifuge of blood at 3000 rpm and 4 °C for 15 min. Serum samples were stored at −80 °C until further analysis. Serum triglyceride (TG), total cholesterol (TC), low-density lipoprotein cholesterol (LDL), high-density lipoprotein cholesterol (HDL), alanine aminotransferase (ALT), and aspartate aminotransferase (AST) were measured by commercially available kits (Nanjing Jiancheng Bioengineering Institute, Nanjing, China). All assays were used following the manufacturer’s instructions.

### 2.7. Measurement of Liver Triglyceride (TG)

The level of liver TG was determined by using a commercial assay (Nanjing Jiancheng Bioengineering Institute, Nanjing, China). Each step of the experimental operation was completed according to the manufacturer’s guidelines.

### 2.8. Quantitative Real-Time PCR Analysis

Total RNA from the liver was extracted using Total RNA Isolation Kit (Beibei Biotech Co., Ltd., Zhengzhou, China), and 1 μg was used for the reverse transcription of total RNA to generate cDNA using HiScript^®^ III RT SuperMix for qPCR kits with gDNA wiper (Vazyme Biotech Co., Ltd., Nanjing, China). Gene expression was measured by quantitative real-time PCR using UltraSYBR Mixture (Applied Biosystems, CWBIO) on a PCR amplification system (Roche LightCycler 480). Primer pairs were designed by using Primer-BLAST software in NCBI and the specificity of primer sequences was confirmed via the BLAST search. The primers were made by Beijing Liuhe BGI Co., Ltd. (Beijing, China). Primer sequence information involved in the experiments is listed in Table S3. We calculated the relative mRNA expression using the 2^−ΔΔCT^ method and used 18S ribosomal RNA as the reference gene.

### 2.9. Western Blot

Total proteins from frozen tissues of mice liver were extracted using the protein extraction kit (Beyotime Biotech, Shanghai, China), and samples were centrifuged at 14,000 rpm for 10 min at 4 °C. Supernatants were then collected, and the protein contents were measured using a BCA Protein Assay Kit (Beyotime Biotech, Shanghai, China). Sample supernatants were mixed into a 5 × SDS-PAGE loading buffer and heated for 10 min at 100 °C for the subsequent electrophoresis loading procedure. Equivalent amounts of samples (10–30 μg of total protein) were loaded into each lane of the Western blot gel and were separated by SDS-PAGE, followed by electro-transferred onto PVDF membranes (Millipore, Billerica, MA, USA) using a wet transfer method. Membranes were later blocked with 5% skimmed milk powder diluted in TBST solution for 2 h at room temperature. Membranes were incubated with specific primary antibodies [FGF21 (Abcam, Cambridge, UK, 1:1000 dilution), PGC-1α (Proteintech, Wuhan, China, 1:5000 dilution), CPT1A (Proteintech, Wuhan, China, 1:5000 dilution), SCD1 (ABclonal, Wuhan, China, 1:2000), β-actin (Proteintech, Wuhan, China, 1:5000 dilution)] at 4 °C overnight. On the following day, the blots were incubated with HRP-conjugated goat anti-rabbit or anti-mouse IgG (Proteintech, Wuhan, China, 1:5000 dilution) secondary antibodies for 1.5 h at room temperature. Protein bands were detected with an ECL advanced kit using GelView 6000Plus Image Analysis System (BLT Biotech, Guangzhou, China). The relative abundance of bands was analyzed with ImageJ software.

### 2.10. Statistical Analysis

All results are presented as mean ± SEM. A two-way ANOVA was used to evaluate the differences between groups involved in two intervention factors (diet × exercise). Bonferroni’s post hoc comparison tests were reported only if interaction effects were statistically significant. An unpaired two-tailed Student’s *t*-test was used to assess the statistical significance between the two groups. Data analyses were performed using SPSS 25.0 and GraphPad Prism 8.0 software. Statistical significance was set at a level of *p* < 0.05.

## 3. Results

### 3.1. Ketogenic Diet and Ketogenic Diet Combined with Aerobic Exercise Decrease Body Weight in Obese Mice

**Figure 1:**
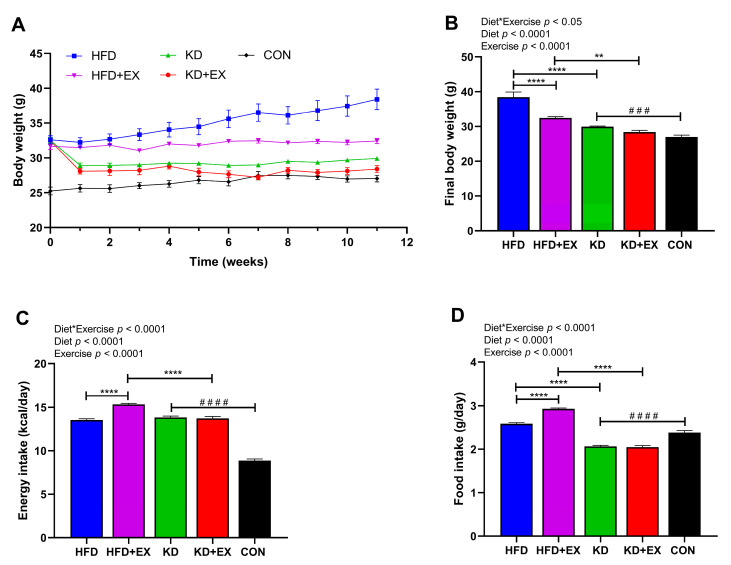
Body weight change and energy intake over the 11-week dietary and aerobic exercise intervention in C57BL/6J obese mice. (**A**) Body weight change of HFD, HFD + EX, KD, KD + EX groups of mice. (**B**) Final body weight of HFD, HFD + EX, KD, and KD + EX groups of mice. (**C**) Average energy intake of each group. (**D**) Average food intake of each group. Values are given as mean ± SEM, n = 8. For panels (**B**–**D**), *p* values from the two-way ANOVA (i.e., main diet and exercise effects and diet*exercise interactions) are presented in the top left corner; Bonferroni post hoc test indicated significant differences (identified with an asterisk) at the following *p* values, ** *p* < 0.01; **** *p* < 0.0001; unpaired two-tailed Student’s *t*-test was used to indicate statistical significance between the KD and CON groups at the following *p* values, ### *p* < 0.001, #### *p* < 0.0001.

To determine the effect of KD and KD combined with exercise on weight loss in obese mice, the changes in body weight during the 11-week intervention, the final body weight, and energy intake were recorded (Figure 1). As shown in Figure 1A, there was no significant difference in the baseline body weight among four obese groups (in grams): HFD (32.60 ± 0.58), HFD + EX (31.67 ± 0.46), KD (32.53 ± 0.45), KD + EX (32.45 ± 0.53) (*p* > 0.05). While the HFD-fed sedentary mice continued to gain weight, mice in the KD-fed group maintained their body weight after the initial drop in their weight during the first week of the dietary intervention (Figure 1A). An interaction effect between diet and exercise was observed in the final body weight (*p* < 0.05) (Figure 1B). KD-fed mice had significantly lower final body weight than HFD-fed mice in sedentary groups (*p* < 0.0001), and the same was true in the exercise training groups (*p* < 0.01) (Figure 1B). Moreover, the final body weight of the exercise training group was significantly lower than that of the sedentary group in HFD-fed mice (*p* < 0.0001), but there was no significant difference in final body weight between the exercise and sedentary KD-fed groups (*p* > 0.05) (Figure 1B). Furthermore, compared with the CON group, the KD-fed sedentary group mice had higher final body weight (*p* < 0.001) (Figure 1B). There was a significant interaction effect in energy intake (*p* < 0.0001), which was higher in the HFD-fed exercise group compared with the HFD-fed sedentary group (*p* < 0.0001), while the KD-fed exercise group had significantly lower energy intake than the HFD-fed exercise group (*p* < 0.0001) (Figure 1C). Interestingly, despite two different dietary interventions, there was no significant difference in energy intake between sedentary groups (*p* > 0.05), which shows that despite having similar energy intake, KD was effective in reducing body weight, and neither was there a significant difference between the KD-fed groups (*p* > 0.05) (Figure 1C). In addition, KD-fed sedentary group had significantly higher energy intake than the CON group (Figure 1C), which indicates mice had satisfactory palatability for KD. As shown in Figure 1D, exercise was associated with increased food intake in HFD-fed groups (*p* < 0.0001). Additionally, KD significantly reduced food intake compared with HFD-fed groups (*p* < 0.0001), and the KD-fed sedentary group had lower food intake than that in the CON group (*p* < 0.0001) (Figure 1D), although the energy intake (kcal/day) was higher than the CON group (Figure 1C). These results suggest that the KD intervention decreased body weight, but there was no combined effect of KD and aerobic exercise on weight loss.

### 3.2. Ketogenic Diet Feeding Enhances Energy Expenditure and Fat Oxidation

**Figure 2:**
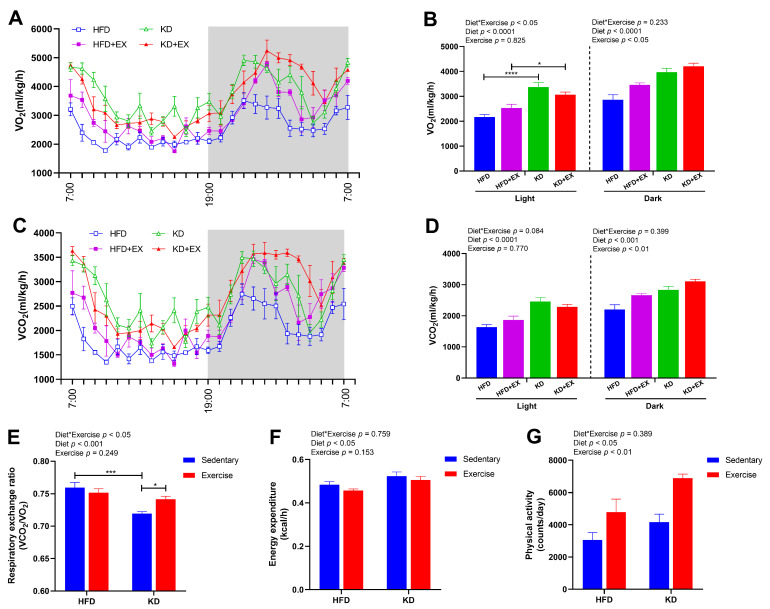
Energy metabolism in HFD, HFD + EX, KD, KD + EX groups of mice. (**A**) and (**B**) O_2_ consumption (VO_2_). (**C**) and (**D**) CO_2_ production (VCO_2_). (**E**) Respiratory exchange ratio (VCO_2_/VO_2_). (**F**) Energy expenditure. (**G**) Physical activity. Values are given as mean ± SEM, n = 3–4. For panels B, D, E and F, *p* values from the two-way ANOVA (i.e., main diet and exercise effects and diet*exercise interactions) are presented in the top left corner; Bonferroni post hoc test indicated significant differences (identified with an asterisk) at the following *p* values, * *p* < 0.05; *** *p* < 0.001; **** *p* < 0.0001.

To explore the effects of KD and KD combined with exercise on energy expenditure in obese mice, we performed metabolic cage studies on these subsets of animals. As shown in Figure 2A,B; there was an interaction effect in oxygen consumption during the 12 h light cycle (*p* < 0.05). Oxygen consumption was significantly increased in the KD-fed sedentary group relative to the HFD-fed sedentary group (*p* < 0.0001), and it was also enhanced in the KD-fed exercise group compared with the HFD-fed exercise group (*p* < 0.05) (Figure 2A,B). During the 12 h dark cycle, the main effects of diet and exercise were observed, indicating KD feeding (*p* < 0.0001) and exercise (*p* < 0.05) both increased the oxygen consumption, but there was no significant interaction (Figure 2A,B). As shown in Figure 2C,D; KD was associated with enhanced carbon dioxide production (main effect) during the 12 h light cycle (*p* < 0.0001) and during the 12 h dark cycle (*p* < 0.001). In contrast, exercise had a main effect showing higher carbon dioxide production (*p* < 0.01) during the 12 h dark cycle (Figure 2C,D). As shown in Figure 2E, an interaction effect was observed in RER (*p* < 0.05), which was lower in the KD-fed sedentary group compared with the HFD-fed sedentary group (*p* < 0.001), with the KD-fed mice being close to an RER of 0.7, indicating almost complete reliance on lipids for oxidative metabolism. Additionally, exercise enhanced the RER in KD-fed groups (*p* < 0.05) indicating a possible increase in the utilization of amino acids for gluconeogenesis (Figure 2E). A main effect of diet was found for resting energy expenditure (*p* < 0.05), indicating that KD feeding significantly increased energy expenditure (Figure 2F). Main effects of both diet (*p* < 0.05) and exercise (*p* < 0.01) were found for the physical activity, showing KD feeding and exercise training both elevated the levels of voluntary wheel running (Figure 2G). Together these findings indicate that KD and KD combined with aerobic exercise both increased energy expenditure and enhanced the proportion of fat oxidized as a fuel. This increase in energy expenditure explains why KD-fed mice did not gain weight despite consuming almost the same energy as HFD-fed mice.

### 3.3. Ketogenic Diet and Ketogenic Diet Combined with Aerobic Exercise Effectively Promote Fat Loss

**Figure 3:**
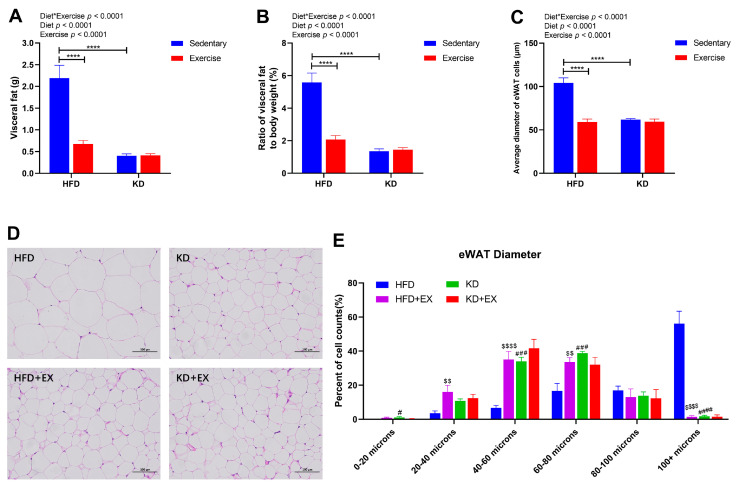
Visceral fat masses and eWAT Diameters in four treatment groups of mice. (**A**) Visceral fat masses (n = 8). (**B**) Ratio of visceral fat to body weight (%) (n = 8). (**C**) Average diameter of eWAT cells (n = 5). (**D**) Representative HE staining of eWAT sections (Scale bars = 100 μm; Original magnification, 200×) (n = 5). (**E**) eWAT diameter range (n = 5). Data are presented as mean ± SEM. For panels A, B and C, *p* values from the two-way ANOVA (i.e., main diet and exercise effects and diet*exercise interactions) are presented in the top left corner; Bonferroni post hoc test indicated significant differences (identified with an asterisk) at the following *p* values, **** *p* < 0.0001. For panel E, # indicates the KD group is statistically different from the HFD group at the following *p* values, # *p* < 0.05, ### *p* < 0.001, #### *p* < 0.0001; $ indicates the HFD + EX group is statistically different from the HFD group at the following *p* values, $$ *p* < 0.01, $$$$ *p* < 0.0001.

A strong interaction effect between diet and exercise was observed in the mass of visceral fat (*p* < 0.0001) (Figure 3A). The average visceral fat mass in the sedentary HFD-fed group was 5.44-fold that of the sedentary KD-fed group, and 3.25-fold that of the HFD-fed exercise group (Figure 3A). KD and aerobic exercise intervention both led to significantly lower mass of visceral fat (*p* < 0.0001), but there was no significant difference between KD-fed groups (*p* > 0.05) (Figure 3A). The treatment differences for the ratio of visceral fat to body weight were similar to that observed for visceral fat (Figure 3B). As shown in the histology data in Figure 3C,D; the average diameter of eWAT cells was significantly reduced after exercise in the HFD-fed group (*p* < 0.0001) and in the sedentary KD-fed group (*p* < 0.0001) (interaction effect). There was, however, no significant effect of exercise in the KD-fed mice (Figure 3C). In addition, histological analysis of the distribution ratio of the eWAT diameter range is shown in Figure 3E. An interaction effect was observed in eWAT diameter between 0–20 μm (*p* < 0.05), 20–40 μm (*p* < 0.05), 40–60 μm (*p* < 0.05), 60–80 μm (*p* < 0.01), and greater than 100 μm (*p* < 0.0001) (Figure 3E). KD feeding and exercise training both significantly increased the percent of cell counts for eWAT diameter less than 80 μm while reducing that in eWAT diameter greater than 100 μm compared with the HFD-fed sedentary group (Figure 3E). These results suggest that KD intervention and exercise training could both reduce the visceral fat mass, the proportion of visceral fat to body weight, and eWAT cell diameters, but KD-fed combined with the exercise group did not show an additive effect on adiposity.

### 3.4. A Combination of Ketogenic Diet and Aerobic Exercise Shows Additive Metabolic Effects on Serum Biomarkers

**Figure 4:**
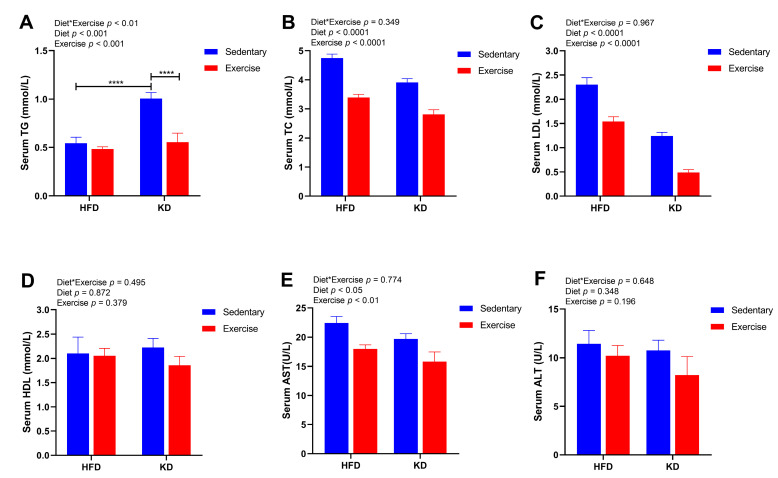
Effects of KD intervention and KD combined with exercise on serum lipid metabolism and liver injury in each group. (**A**) TG, (**B**) TC, (**C**) LDL, (**D**) HDL, (**E**) AST and (**F**) ALT in serum. Data are presented as mean ± SEM, n = 6–8. For panels A–F, *p* values from the two-way ANOVA (i.e., main diet and exercise effects and diet*exercise interactions) are presented in the top left corner; Bonferroni post hoc test indicated significant differences (identified with an asterisk) at the following *p* values, **** *p* < 0.0001.

To further determine the effects of KD intervention and KD combined with exercise on circulating markers of lipid metabolism and liver injury, we measured the levels of serum TG, TC, LDL, HDL, ALT and AST. Although KD intervention significantly increased the level of serum TG in sedentary mice (*p* < 0.0001), exercise significantly ameliorated this effect by reducing TG levels in KD mice (*p* < 0.0001) (Figure 4A). In addition, the main effects of diet and exercise were both observed in the levels of TC and LDL (Figure 4B,C). Both TC and LDL levels declined with KD and with aerobic exercise (*p* < 0.0001) (Figure 4B,C). Interestingly, a main effect of diet was observed for the levels of AST (*p* < 0.05), indicating lower AST in KD-fed mice (Figure 4E). Aerobic exercise training resulted in a main effect on the levels of AST (lower) (*p* < 0.01) (Figure 4E). However, there were no significant differences in the levels of HDL and ALT (*p* > 0.05) (Figure 4D,F). These results indicate that KD combined with aerobic exercise effectively decreased the levels of serum lipids and may improve liver injury-related indicators to a certain degree.

### 3.5. Ketogenic Diet and Ketogenic Diet Combined with Aerobic Exercise Ameliorate Hepatic Physiological State

**Figure 5:**
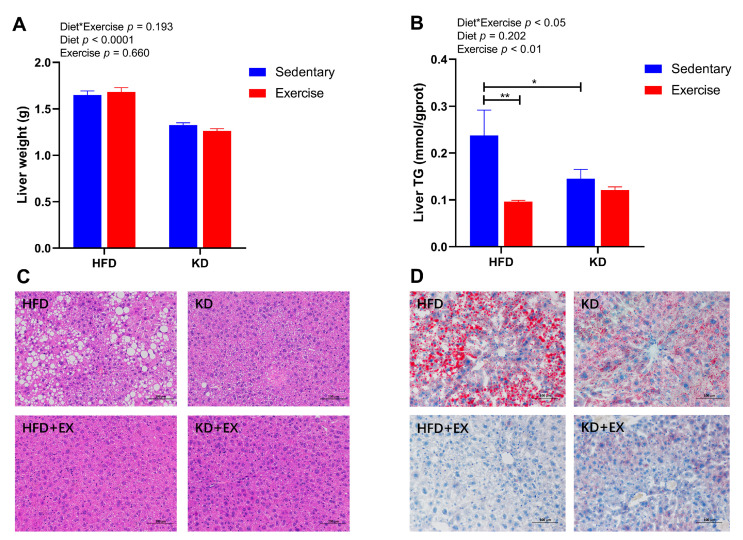
Effects of KD intervention and KD combined with exercise on hepatic lipid accumulation and hepatic steatosis. (**A**) Liver weight (n = 8). (**B**) Quantification of liver TG; (**C**) Representative images of HE staining of liver sections (Scale bars = 100 μm; Original magnification, 200×); (**D**) Representative images of Oil-red O staining of liver sections (Scale bars = 100 μm; Original magnification, 200×) (n = 5–6). Data are presented as mean ± SEM. For panels A and B, *p* values from the two-way ANOVA (i.e., main diet and exercise effects and diet*exercise interactions) are presented in the top left corner; Bonferroni post hoc test indicated significant differences (identified with an asterisk) at the following *p* values, * *p* < 0.05; ** *p* < 0.01.

Considering the potential adverse effects of KD on hepatic physiology, we further investigated the liver weight, the levels of liver triglyceride, and histological analysis by H&E staining and Oil-red O staining. A main effect of diet was observed for the liver weight (Figure 5A), indicating lower liver weight in KD-fed mice (*p* < 0.0001). In contrast, an interaction effect was detected in the levels of liver TG (*p* < 0.05) (Figure 5B). KD significantly decreased the levels of liver TG in the sedentary group (*p* < 0.05), and exercise also lowered TG but only in HFD-fed mice (*p* < 0.01) (Figure 5B). As shown in Figure 5C, H&E staining showed that the sedentary HFD-fed group had more mixed micro- and macrovesicular steatosis than the other three groups. However, after 8-week of aerobic exercise training or KD intervention, hepatic steatosis was largely attenuated in comparison with the sedentary HFD-fed group (Figure 5C). Similarly, Oil-red O staining revealed large numbers of lipid droplets in the sedentary HFD group (Figure 5D). However, the KD-fed intervention reduced fat droplets in the livers (Figure 5D). Moreover, the exercise training groups showed even fewer and smaller deposits of lipids in hepatocytes than the sedentary groups (Figure 5D). Together, these findings suggest that contrary to HFD-feeding, KD-feeding significantly lowers liver weight, circulating TG, hepatic steatosis, and lipid accumulation. Additionally, aerobic exercise further reduces lipid storage in the liver.

### 3.6. Ketogenic Diet Combined with Aerobic Exercise Modulates Lipogenesis and Lipolysis-Related Gene Expression in Liver

**Figure 6:**
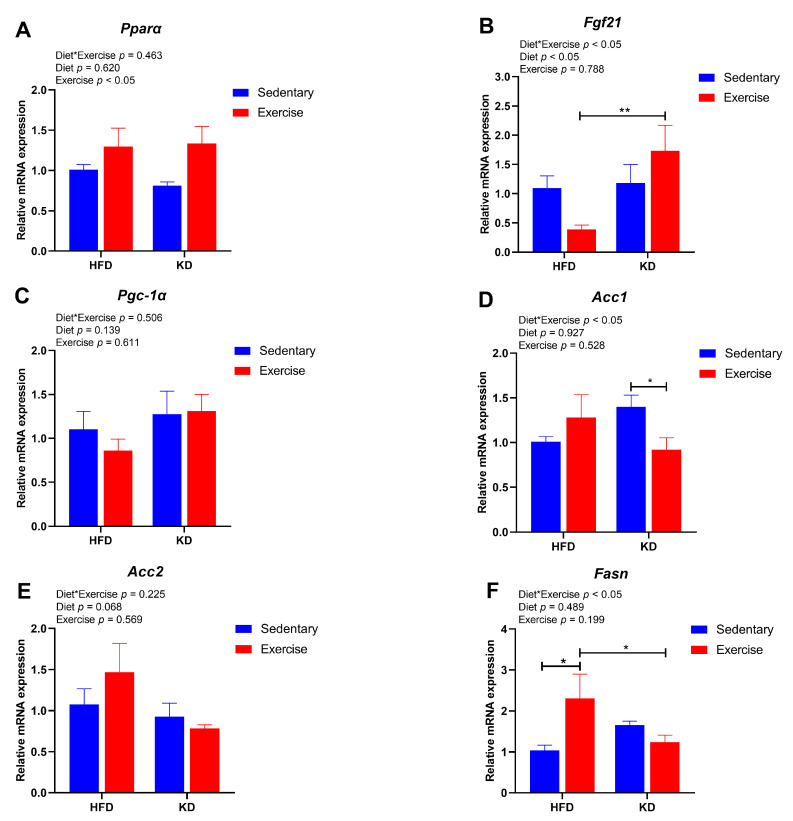
Effects of KD intervention and KD combined with exercise on the fatty acid oxidation and synthesis-related gene expression in livers. Gene expression of (**A**) *Pparα*, (**B**) *Fgf21* and (**C**) *Pgc-1α*, related to fatty acid oxidation in each group of mouse livers. Gene expression of (**D**) *Acc1*, (**E**) *Acc2* and (**F**) *Fasn*, related to fatty acid synthesis in four group mouse livers. Data are presented as mean ± SEM, n = 6. For panels (**A**–**F**), *p* values from the two-way ANOVA (i.e., main diet and exercise effects and diet*exercise interactions) are presented in the top left corner; Bonferroni post hoc test indicated significant differences (identified with an asterisk) at the following *p* values, * *p* < 0.05; ** *p* < 0.01.

Next, we further explored the effects of KD and KD combined with aerobic exercise on the gene expression of hepatic lipid metabolism. Therefore, we tested the expression of genes related to fatty acid metabolism, including *Pparα*, *Fgf21*, *Pgc-1α*, *Acc1*, *Acc2* and *Fasn*. Exercise had a main effect showing slightly higher gene expression of *Pparα* (*p* < 0.05) (Figure 6A). An interaction effect was found for the *Fgf21* (*p* < 0.05), in which KD-fed exercised mice had higher expression of *Fgf21* than HFD-fed exercised mice (*p* < 0.01) (Figure 6B). However, there were no main effects or an interaction effect detected in *Pgc-1α* (*p* > 0.05) (Figure 6C). As for fatty acid synthesis-related genes, a significant interaction effect between diet and exercise was found for the *Acc1* gene (*p* < 0.05) and *Fasn* gene (*p* < 0.05) (Figure 6D,F). Aerobic exercise decreased the gene expression of *Acc1* in KD-fed groups (*p* < 0.05) (Figure 6D). It showed a pattern of increase with exercise in HFD-fed mice but decreased in KD-fed mice in *Fasn* (*p* < 0.05) (Figure 6F). However, there was no significant difference in the gene expression of *Acc2* (*p* > 0.05) (Figure 6E). Overall, this evidence indicates that KD combined with exercise shows a better effect on lowering the expression levels of lipogenesis-related genes than the intervention of KD without exercise.

### 3.7. Ketogenic Diet Combined with Aerobic Exercise Modulates Lipogenesis and Lipolysis-Related Protein Expression in Liver

**Figure 7:**
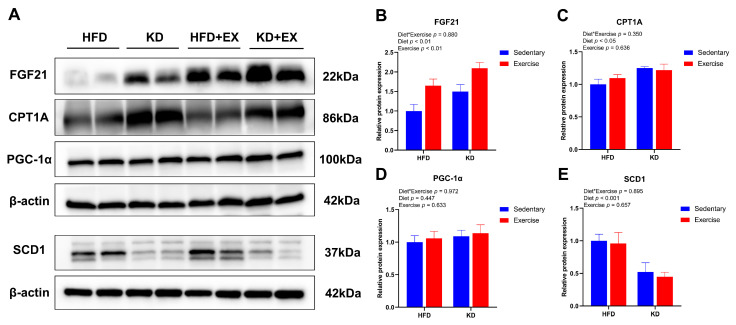
Effects of KD intervention and KD combined with exercise on the fatty acid oxidation and synthesis-related protein expression in each group of mouse livers. (**A**) Representative western blots. Protein quantification analysis of (**B**) FGF21, (**C**) CPT1A, (**D**) PGC-1α and (**E**) SCD1 in livers. Data are presented as mean ± SEM, n = 6. For panels B–E, *p* values from the two-way ANOVA (i.e., main diet and exercise effects and diet*exercise interactions) are presented in the top left corner.

To further investigate the effects of KD and KD combined with aerobic exercise on the protein expression of fatty acid metabolism in the mouse livers, we tested several biomarkers of protein expression related to lipid metabolism, including FGF21, PGC-1α, CPT1A and SCD1. The main effect of diet and exercise were both observed in the expression of FGF21 (*p* < 0.01), showing that KD and aerobic exercise enhanced its relative expression (Figure 7A,B). Diet had a main effect on CPT1A protein expression (*p* < 0.05), indicating higher relative expression levels in KD-fed mice (Figure 7A,C). However, there was no significant difference in PGC-1α (*p* > 0.05) (Figure 7A,D). Furthermore, KD resulted in a main effect on SCD1 protein expression (*p* < 0.05), suggesting lower protein levels of lipogenic factors in KD-fed mice (Figure 7A,E). This is consistent with the reports suggesting that increased intake of dietary fat reduces de novo lipogenesis in the liver [32,33]. Taken together, these results indicate that KD feeding could modulate the expression levels of lipid metabolism-related proteins, and aerobic exercise could further enhance lipolysis-related protein levels.

## 4. Discussion

Emerging evidence suggests that ketogenic diets can reduce body fat stores and might be used as a treatment for weight loss in obesity [34,35,36]. However, the KD intervention applied to weight loss in obesity may have some adverse effects [16,21]. The purpose of this study was to examine the effects of KD combined with aerobic exercise on weight loss, visceral fat composition, blood parameters, and hepatic lipid metabolism in obese mice. For comparison, we also studied the effects of a typical obesogenic rodent HFD with or without exercise. We found that compared with HFD-fed mice, feeding a KD to mice led to lower body weight, reduced adiposity, and improved hepatic health. This was accompanied by greater fat oxidation and increased energy expenditure in KD-fed vs. HFD-fed mice. Importantly, aerobic exercise markedly improved metabolic health markers in HFD-fed mice but had minimal effects on body weight and adiposity in KD-fed mice. However, exercise decreased the levels of serum TG, TC, LDL and AST compared with the sedentary KD-fed mice.

This study utilized a classic rodent ketogenic diet (90% energy as fat, 10% energy as protein), and we found that mice tolerated the KD well, consuming at least as many calories as mice fed the high-fat diet in sedentary groups and even greater energy intake than the chow-fed mice. Interestingly, contrary to HFD-feeding, mice eating KD did not gain weight despite the higher energy density (6.7 vs. 5.24 kcal/g) and lower protein (9.99 vs. 20% energy), both of which are associated with increased energy intake in non-obese mice [37]. This could be due to the inhibitory effects of ketones on appetite, which could have attenuated the increase in daily calorie intake on a protein-diluted KD [38]. Despite ingesting the same calories as HFD-fed mice, mice on KD had lower body weight and adiposity. This could be due to a combination of greater energy expenditure, and increased utilization of fats for oxidative metabolism. These findings correspond well with previous rodent literature [13], which reported that KD feeding increased energy expenditure and lowered respiratory quotient indicating higher fat oxidation. In addition, the previous study found that a 12-week ketogenic diet intervention reduced total body fat compared with mice fed a high-fat diet [13]. Furthermore, many studies have demonstrated the effectiveness of KD in reducing both blood glucose and insulin levels which increases energy storage in adipose tissue and stimulates lipogenesis in the liver [39,40]. Therefore, we speculate that lower circulating insulin levels may be an underlying factor of the metabolic effects of KD.

We found significant improvements in several markers of metabolic health with aerobic exercise in HFD-fed mice. However, in KD-fed mice, exercise did not lead to substantial changes in body weight or adiposity. This could be because energy expenditure and fat oxidation were already high, hepatic fat content was reduced in KD-fed mice, and exercise could not cause additional improvements. One of the negative effects of KD was a significantly increased level of TG in serum, which is consistent with the findings of others [16,21], and was even higher than the HFD-fed group. In contrast, liver TG content was higher in the sedentary HFD-fed group than in the sedentary KD-fed mice. A possible explanation is the reduced hepatic expression in the HFD-fed mice of apolipoproteins (especially Apo-B) that export triglyceride-rich VLDL particles out of the liver and into the circulation. Hepatic TG content is influenced by fatty acids derived from the diet as well as de novo hepatic lipogenesis driven by dietary carbohydrates [41,42]. Importantly, HFD had higher carbohydrate content than KD. Therefore, we speculate that the higher liver TG and lower serum TG levels in HFD-fed sedentary mice than the KD-fed sedentary mice are probably because HFD-fed mice are synthesizing more TG in the liver than they can export into circulation via the apolipoprotein-VLDL system. This reflects a possible combination in HFD-fed mice of increased hepatic de novo lipogenesis, decreased hepatic fat oxidation, and reduced expression of apolipoproteins in HFD-fed mice. Dietary studies in rodents have shown that very low protein (approximately 5% protein) diets lead to a similar phenotype where animals have increased liver TG content, decreased expression of hepatic apolipoproteins, and reduced circulating TG levels [41,43]. The diets used in our study were not very low in protein content, but it is possible that a HFD may lead to significantly lower hepatic expression of apolipoproteins than a KD. To this end, evidence in children suggests that a KD significantly increases the levels of apolipoprotein-B-containing lipoprotein particles in circulation [44].

Remarkably, aerobic exercise was associated with a reduction in serum TG relative to the sedentary KD group. However, in contrast to previous research [21,45], we did not observe adverse responses caused by KD in other serum lipid biomarkers. A possible reason is the previous research used a different mouse model, namely, type 2 diabetic mice, and different metabolic states may have led to distinct responses to KD feeding. Indeed, previous rodent literature have also reported that KD decreased serum lipids by inducing a reduction in insulin [13]. In addition, KD combined with aerobic exercise was associated with lowered serum TC, LDL and AST compared with the sedentary KD treatment, indicating that aerobic exercise combined with KD intervention could have health benefits in improving these aspects.

At a molecular level, the peroxisome proliferator-activated receptor α (PPARα)-fibroblast growth factor 21 (FGF21) axis could be an important mediator of the metabolic effects of KD and exercise. In the liver, PPARα plays a key role in regulating lipid metabolism, especially during starvation, by promoting fatty acid oxidation and ketogenesis [46,47]. As an important endocrine regulator with hormonal effects predominantly expressed in the liver [48], FGF21 contributes to ketogenesis and ameliorates obesity [49,50]. FGF21 is also one of the exercise-induced hepatokine, and exercise could improve lipid metabolism by affecting the expression level and activity of FGF21 [51,52]. Considered an upstream regulator of FGF21, PPARα could induce hepatic FGF21 expression in rats [53,54]. Our study indicated that exercise enhanced the gene expression of *Pparα* in the two different dietary groups. We also observed the induction of *Fgf21* expression by KD. Since the KD diet had lower protein than the HFD diet, this is consistent with the now well-established role of FGF21 as an endocrine signal activated by protein restriction [55]. Interestingly, aerobic exercise increased the level of FGF21 protein expression in HFD-fed groups, which is also in line with a previous study [56]. Additionally, KD feeding and aerobic exercise both enhanced the protein expression levels of FGF21. Higher FGF21 protein expression levels in the KD would facilitate hepatic lipid oxidation [53], which may be the reason for the better hepatic physiological state. KD could increase FGF21 liver expression, and this may be responsible for the increased energy expenditure in KD groups, as injection of recombinant FGF21 increases energy expenditure in obese mice [57].

Contrary to the increased expression of FGF21, KD alone or in combination with exercise resulted in an overall reduction in the expression of the factors involved in de novo lipogenesis. For example, KD combined with exercise decreased the level of *Acc1* gene expression compared with KD intervention without exercise. Furthermore, as a key metabolic factor that promotes hepatic triglyceride synthesis, we found that KD feeding decreased the protein expression levels of SCD1. Similarly, the expression of the lipogenic gene *Fasn* was lower in the KD-fed exercised group than HFD-fed exercised group. These results are consistent with previous findings showing that FGF21 plays a prominent effect in decreasing de novo triglyceride biosynthesis and reducing hepatic triglyceride content in experimental animals [57,58]. Moreover, since the dietary carbohydrate-insulin axis is the major activator of de novo lipogenesis [59], the reduced expression of lipogenic factors was expected because of the absence of carbohydrates in our KD.

This research is not without limitations. C57BL/6J mice were used in this study because they are prone to metabolic disorders induced by a high-fat diet. However, this study only involved one model animal, and as such, we cannot preclude differences in metabolic responses to this intervention in other species including humans or other strains of mice. The second limitation is that only one type of exercise program was included. Low- to moderate-intensity aerobic exercise has always been regarded as a traditional and effective way to combat obesity. In this study, the combination of KD and moderate aerobic exercise did not produce a synergistic effect on weight and fat loss. Exercise intensity, volume, duration, intervention period, and training types are all factors that impact the improvement of metabolic effects. Other forms of exercise, such as resistance exercise, and high-intensity interval training (HIIT) can also be taken into account in an obesity management program [60,61]. Thus, further studies could examine whether there is a more efficient exercise program than aerobic exercise included in this joint intervention for obesity.

## 5. Conclusions

In conclusion, the results of our current study suggest that both the ketogenic diet and ketogenic diet combined with moderate aerobic exercise reduced body weight and fat mass, but KD combined with exercise had an advantage over KD intervention without exercise in ameliorating serum biomarkers, hepatic steatosis and liver lipid metabolism in high-fat diet-induced obese mice. These findings could lead to a novel therapeutic strategy for the management of obesity.

## Data Availability

The data that support the findings of this research are available from the corresponding author upon reasonable request.

## Supplementary Materials

The following supporting information can be downloaded at: https://www.mdpi.com/article/10.3390/nu15010251/s1, Table S1: Percentage of three macronutrient composition in diet; Table S2: Detailed description of the dietary composition; Table S3: qPCR primer sequences.
