# Supplementary material for: Ketogenic Diet Combined with Moderate Aerobic Exercise Training Ameliorates White Adipose Tissue Mass, Serum Biomarkers, and Hepatic Lipid Metabolism in High-Fat Diet-Induced Obese Mice
Source: Nutrients. 2023 Jan 3;15(1):251. doi: 10.3390/nu15010251 (PMC9823610; doi:10.3390/nu15010251)
Supplement: Supplementary file 1 [file nutrients-15-00251-s001.zip › nutrients-2108619-supplementary.docx]

**Table S1.** Percentage of three macronutrient composition in diet

| **Dietary composition** | **Chow**  **(AIN-93G)** | **HFD**  **(H10060)** | **KD**  **(XTKD01)** |
| --- | --- | --- | --- |
| Protein (g%) | 20 | 26 | 16.63 |
| Carbohydrate (g%) | 64 | 26 | 0 |
| Fat (g%) | 7 | 35 | 66.5 |
| Protein (kcal%) | 20.3 | 20 | 9.99 |
| Carbohydrate (kcal%) | 63.9 | 20 | 0 |
| Fat (kcal%) | 15.8 | 60 | 89.91 |
| Total (kcal%)  kcal/g | 100 | 100 | 100 |
|  | 3.9 | 5.24 | 6.7 |

Sources of macronutrients for each diet are as follows: chow diet—carbohydrate: corn starch, maltodextrin, and sucrose; protein: casein and cystine; fat: soybean oil; high-fat diet (HFD)—carbohydrate: maltodextrin and sucrose; protein: casein and cystine; fat: soybean oil and lard; ketogenic diet (KD)—protein: casein and L-cystine; fat: soybean oil and cocoa butter.

**Table S2.** Detailed description of the dietary composition

|  | **Chow**  **(AIN-93G)** | | **HFD**  **(H10060)** | | **KD**  **(XTKD01)** | |
| --- | --- | --- | --- | --- | --- | --- |
| **Ingredient** | **g** | **kcal** | **g** | **kcal** | **g** | **kcal** |
| Casein | 200 | 800 | 258.45 | 1033.8 | 100 | 400 |
| L-Cystine | 3 | 12 | 3.88 | 15.52 | 1.5 | 6 |
| Corn Starch | 397 | 1588 | 0 | 0 | 0 | 0 |
| Maltodextrin | 132 | 528 | 161.53 | 646.12 | 0 | 0 |
| Sucrose | 100 | 400 | 88.91 | 355.64 | 0 | 0 |
| Cellulose | 50 | 0 | 64.61 | 0 | 50 | 0 |
| Soybean Oil | 70 | 630 | 32.31 | 290.79 | 25 | 225 |
| Lard | 0 | 0 | 316.6 | 2849.4 | 0 | 0 |
| Cocoa Butter | 0 | 0 | 0 | 0 | 381 | 3429 |
| Mineral Mix | 35 | 0 | 12.92 | 0 | 50 | 0 |
| Vitamin Mix | 10 | 40 | 12.92 | 51.68 | 1 | 4 |
| Choline Bitartrate | 2.5 | 0 | 2.58 | 0 | 2 | 0 |
| Antioxidant TBHQ | 0.014 | 0 | 0 | 0 | 0 | 0 |
| Calcium hydrogen phosphate | 0 | 0 | 16.8 | 0 | 0 | 0 |
| Calcium Carbonate | 0 | 0 | 7.11 | 0 | 0 | 0 |
| Potassium Citrate | 0 | 0 | 21.32 | 0 | 0 | 0 |
| FD&C Blue Dye #1 | 0 | 0 | 0.065 | 0 | 0 | 0 |
| FD&C Red Dye #40 | 0 | 0 | 0 | 0 | 0.025 | 0 |
| FD&C Yellow Dye #5 | 0 | 0 | 0 | 0 | 0.025 | 0 |
| Total | 1000 | 3998 | 1000 | 5242.95 | 610.55 | 4064 |

**Table S3.** qPCR primer sequences

| **Gene** | **Forward primer sequence (5’ to 3’)** | **Reverse primer sequence (5’ to 3’)** |
| --- | --- | --- |
| *Fasn* | GGCCCCTCTGTTAATTGGCT | GGATCTCAGGGTTGGGGTTG |
| *Acc1* | CTAAACCAGCACTCCCGAT | TGAAAGGCCAAACCATCCTG |
| *Acc2* | ATGATCGCAGATGTCAACCT | TTCAAAAGGAATGGGGGTCA |
| *Pparα* | AACTATTCGGCTGAAGCTGG | CTTGGCATTCTTCCAAAGCG |
| *Fgf21* | CGCAGTCCAGAAAGTCTCC | ATCAAAGTGAGGCGATCCAT |
| *Pgc-1α* | CTTCGGTCATCCCTGTCAAG | ATCCCAAGGGTAGCTCAGTT |
| *18S rRNA* | GTAACCCGTTGAACCCCATT | CCATCCAATCGGTAGTAGCG |

*Fasn*, fatty acid synthase; *Acc1*, acetyl-CoA carboxylase 1; *Acc2*, acetyl-CoA carboxylase 2; *Pparα*, peroxisome proliferator activated receptor alpha; *Fgf21*, fibroblast growth factor 21; *Pgc-1α*, peroxisome proliferator-activated receptor gamma coactivator 1 alpha; *18S rRNA*, 18S ribosomal RNA
